# Design, synthesis, and biological evaluation of novel iso-flavones derivatives as H_3_R antagonists

**DOI:** 10.1080/14756366.2018.1509212

**Published:** 2018-10-07

**Authors:** Jian Xin, Min Hu, Qian Liu, Tian Tai Zhang, Dong Mei Wang, Song Wu

**Affiliations:** a State Key Laboratory of Bioactive Substance and Function of Natural Medicines, Institute of Materia Medica, Chinese Academy of Medical Sciences and Peking Union Medical College, Beijing, China;; b School of Pharmacy, Inner Mongolia Medical University, Hohhot, China

**Keywords:** H3R antagonist, iso-flavone, molecular docking

## Abstract

Histamine H_3_ receptor (H_3_R), a kind of G-protein coupled receptor (GPCR), is expressed mainly in the central nervous system (CNS) and plays a vital role in homoeostatic control. This study describes the design and synthesis of a series of novel H_3_R antagonists based on the iso-flavone scaffold. The results of the bioactivity evaluation show that four compounds (**1c**, **2c**, **2h**, and **2o**) possess significant H_3_R inhibitory activities. Molecular docking indicates that a salt bridge, π–π T-shape interactions, and hydrophobic interaction all contribute to the interaction between compound **2h** and H_3_R.

## Introduction

Histamine, a distinctly important neurotransmitter, exerts as a modulator in the brain and dominates several homoeostatic functions such as thermoregulation, fluid balance, and energy metabolism^1^. Apart from that, histamine is also involved in numerous processes, for instance, circadian rhythms, the sleep–wake cycle, attention, memory, learning, and neuroendocrine regulation^2^. According to recent studies, the biosynthesis and release of histamine in central nervous system (CNS) are modulated by four different G-protein coupled receptors (GPCRs) subtypes, namely histamine H_1_ receptor (H_1_R), histamine H_2_ receptor (H_2_R), histamine H_3_ receptor (H_3_R), histamine H_4_ receptor (H_4_R). Unlike H_1_R and H_2_R, H_3_R shows higher homology to H_4_R^3^ and is highly expressed in brain^4^, such as basal ganglia and globus pallidus, which could couple with G i/oα protein and then activate mitogen-activated protein kinase (MAPK) and phosphatidylinositol 3-kinase (PI3K) pathways^5^. Subsequently, the phospholipase A_2_ (PLA_2_) is induced to recruit Ca^2+^ from intracellular stores^6^, reduces cAMP formation^7^, and enhances phosphorylation^2^. Moreover, H_3_R is recognised as an auto- and hetero-receptor on non-histaminergic neurons controlling the release of many other important neurotransmitters^8^^,^^9^, such as acetylcholine, norepinephrine, dopamine, and serotonin^10^. A clinical study revealed that neurotransmitters could trigger the postsynaptic signalling pathways bound to cognition which supported the hypothesis that H_3_R is a drug target for cognitive disorders^3^^,^^6^^,^^11^^,^^12^, especially for Alzheimer Disease (AD), schizophrenia and epilepsy^13–16^. Because of the unique functions of H_3_R, a wide variety of selective H_3_R antagonists have been developed and some of them have shown promising effects^4^^,^^12^^,^^17–21^.

**Figure 1.**
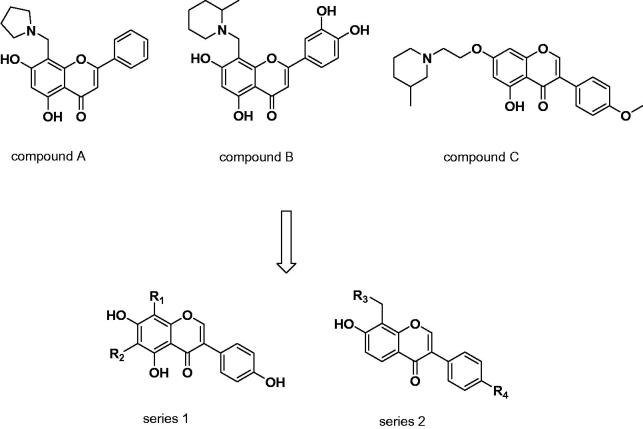
The structures of previously reported H_3_R antagonists and two novel series of compounds.

Flavone and iso-flavone, which are regarded as privileged structures, exhibit variety of pharmacological activities, such as anti-cancer, antimicrobial, anti-inflammatory, and also are used in neurodegenerative disorders, for example, Alzheimer’s disease^22–24^. Our previous study had confirmed the iso-flavone and flavone compounds possessed moderate inhibitory activity against H_3_R^25^. Particularly, the optimization at the 8-position of the flavones and 7-position of iso-flavone provided satisfactory bioactivity (compound **A**, **B,** and **C**, Figure 1), which enlightened us to modify 8-position of iso-flavone to enhance the H_3_R inhibitory effect. In addition, we also want to modify the 6-position of isoflavones to see whether compounds with better antagonistic activity can be obtained. In this current work, two series of novel iso-flavone derivatives were designed and synthesised based on our previous study. After screening the H_3_R inhibitory activities at a fixed concentration, compounds that possessed good H_3_R inhibitory activity were further tested to determine the IC_50_ values. In addition, molecular docking studies were performed to investigate the interaction between H_3_R and the most potent antagonist.

## Materials and methods

### Chemistry

Unless otherwise indicated, all solvents and organic reagents were obtained from commercially available sources and were used without further purification. The reaction process was monitored using thin layer chromatography (TLC) with silica gel plates (thickness = 0.20 mm, GF254) under UV light. Column chromatography was performed using a ZCX-II (200–300 mesh), to purify the final products. All final products were found to have purities ≥95% analysed by HPLC. Melting points were determined using a YRT-3 apparatus (Tian Jin Optical Instrument Factory, Tianjin, China) and were presented as uncorrected values. ^1^H NMR spectra were recorded on a Varian Mercury-300 MHz instrument, whereas ^13 ^C NMR was recorded at 400 MHz on a Varian Mercury using DMSO-d_6_ as a solvent and tetramethylsilane (TMS) as an internal standard (^1^H NMR and ^13 ^C NMR were recorded in different time). Mass spectra were obtained using a Waters Acquity UPLC-SQD mass spectrometer (Waters, Milford, MA). High-resolution mass spectra (HRMS) were recorded on an Agilent Technologies LC/MSD TOF spectrometer (Agilent Technologies Co. Ltd., Santa Clara, CA).

**Scheme 1.**
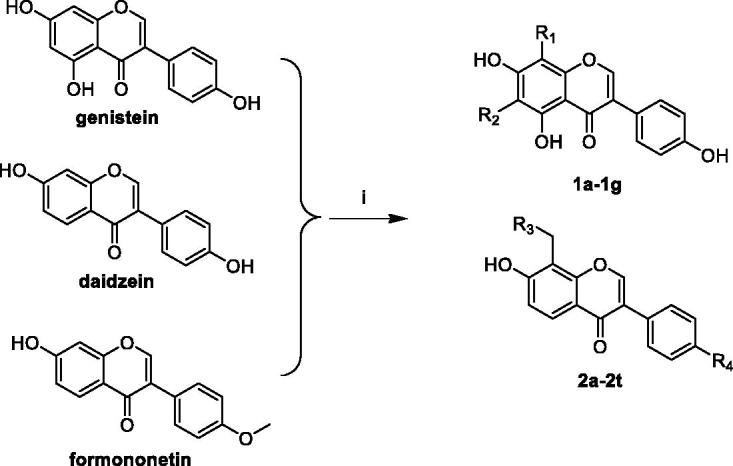
Synthesis of compounds **1a**–**1g**, **2a**–**2t**. Reagents and conditions: (i) 37% formalin, aliphatic amines, 25** **°C, 24 h.

The synthetic route of novel compounds is depicted in Scheme 1. All title compounds were synthesised through Mannich reactions using iso-flavone, 37% formalin, and aliphatic amines as starting materials. Compounds **1a**–**1g**, **2a**–**2i**, and **2j**–**2t** were synthesised from genistein, daidzein, and formononetin, respectively. The use of DMF-methanol as a solvent for formononetin and daidzein never resulted in the formation of 6-substituted products, but only 8-position substituted products were obtained.

### General procedure for the synthesis of compounds 1a–1g

Genistein (0.50 g, 1.85 mmol), 37% formalin (0.30 g, 3.70 mmol), aliphatic amines (0.225 g, 2.780 mmol), and methanol (30 ml) were added into a three-necked flask (100 ml) and stirred at 25 °C for 24 h. After reactions completed monitored by TLC (DCM:MeOH = 10:1), the solvent was removed under reduced pressure. The residue was purified by column chromatography using a mixture of dichloromethane and methanol (30:1) as the eluent to give the target compounds in yields ranging from 41% to 91%.

The similar procedure was followed for the synthesis of compounds **2a–2t**.

Title compounds were characterised as follows:

#### 5,7-dihydroxy-3-(4-hydroxyphenyl)-8-(pyrrolidin-1-ylmethyl)-4H-chromen-4-one (1a)

White solid, yield: 24%; mp 218–220 °C; ^1^H NMR (300 MHz, DMSO-d_6_) *δ* 8.15 (s, 1H), 7.35 (d, *J* = 8.7 Hz, 2H), 6.80 (d, *J* = 8.4 Hz, 2H), 6.10 (s, 1H), 3.96 (s, 2H), 2.83 (m, 4H), 1.83 (m, 4H). ^13 ^C NMR (100 MHz, DMSO-d_6_) *δ* 179.9, 170.4, 161.6, 159.5, 157.8, 153.5, 130.7, 123.8, 122.2, 115.5, 104.7, 102.2, 94.7, 53.3, 50.0, 23.6. HR-MS (ESI) Calcd for C_20_H_19_NO_5_ [M + H]^+^, 354.1341, found: 354.1368.

#### 8-((4-benzylpiperazin-1-yl)methyl)-5,7-dihydroxy-3-(4-hydroxyphenyl)-4H-chromen-4-one (1b)

White solid, yield: 24%; mp 191–193 °C; ^1^H NMR (300 MHz, DMSO-d_6_) *δ* 8.33 (s, 1H), 7.35–7.28 (m, 7H), 6.81 (d, *J* = 8.4 Hz, 2H), 6.17 (s, 1H), 3.81 (s, 2H), 3.46 (s, 2H), 2.56 (m, 4H), 2.40 (m, 4H). ^13 ^C NMR (100 MHz, DMSO-d_6_) *δ* 180.9, 165.5, 161.1, 157.9, 155.6, 154.2, 138.5, 130.7, 129.4, 128.7, 127.5, 122.7, 121.7, 115.6, 104.7, 100.1, 99.5, 62.3, 52.8, 52.5, 51.7. HR-MS (ESI) Calcd for C_27_H_26_N_2_O_5_ [M + H]^+^, 459.1920, found: 459.1939.

#### 5,7-dihydroxy-3-(4-hydroxyphenyl)-8-((3-methylpiperidin-1-yl)methyl)-4H-chromen-4-one (1c)

White solid, yield: 18%; mp 209–211** **°C; ^1^H NMR (300** **MHz, DMSO-d_6_) *δ* 8.29 (s, 1H), 7.36 (d, *J**** ***=*** ***8.7** **Hz, 2H), 6.81 (d, *J**** ***=*** ***8.4** **Hz, 2H), 6.10 (s, 1H), 3.84 (s, 2H), 2.89 (brs, 2H), 2.15 (t, *J**** ***=*** ***10.8** **Hz, 1H), 1.89 (t, *J**** ***=*** ***10.8** **Hz, 1H), 1.65–1.48 (m, 4H), 0.94 (m, 1H), 0.82 (d, *J**** ***=*** ***6.6** **Hz, 3H). ^13^** **C NMR (100** **MHz, DMSO-d_6_) δ 180.4, 168.0, 163.5, 157.9, 157.4, 154.1, 130.7, 122.5, 121.9, 115.6, 104.0, 103.5, 94.5, 60.0, 53.1, 52.7, 32.0, 30.9, 24.9, 19.6. HR-MS (ESI): Calcd for C_22_H_23_NO_5_ [M** **+** **H]^+^, 382.1654, found: 382.1682.

#### 6-(((3R,5S)-3,5-dimethylmorpholino)methyl)-5,7-dihydroxy-3–(4-hydroxyphenyl)-4H-chromen-4-one (1d)

White solid, yield: 20%; mp >250** **°C; ^1^H NMR (300** **MHz, DMSO-d_6_) *δ* 13.02 (s, 1H), 8.36 (s, 1H), 7.37 (d, *J**** ***=*** ***8.4** **Hz, 2H), 6.81 (d, *J**** ***=*** ***8.4** **Hz, 2H), 6.22 (s, 1H), 3.73 (s, 2H), 3.54 (t, *J**** ***=*** ***9** **Hz, 2H), 2.81(d, *J**** ***=*** ***12** **Hz, 2H), 1.78 (t, *J**** ***=*** ***10.8** **Hz, 2H), 1.04 (d, *J**** ***=*** ***6.3** **Hz, 6H). ^13^** **C NMR (100** **MHz, DMSO-d_6_) δ 181.0, 177.9, 176.8, 164.5, 157.9, 155.8, 154.3, 130.7, 121.7, 115.6, 104.9, 100.5, 99.4, 71.5, 58.6, 51.2, 19.4. HR-MS (ESI) Calcd for C_22_H_23_NO_6_ [M** **+** **H]^+^, 39.1604, found: 398.1633.

#### 5,7-dihydroxy-8-((4-(hydroxymethyl)piperidin-1-yl)methyl)-3–(4-hydroxyphenyl)-4H-chromen-4-one (1e)

White solid, yield: 19%; mp 223–225** **°C; ^1^H NMR (300** **MHz, DMSO-d_6_) *δ* 8.29 (s, 1H), 7.36 (d, *J**** ***=*** ***8.4** **Hz, 2H), 6.81 (d, *J**** ***=*** ***8.7** **Hz, 2H), 6.09 (s, 1H), 3.86 (s, 2H), 3.57 (brs, 2H), 2.84 (t, *J**** ***=*** ***6.6** **Hz, 2H), 2.38 (t, *J**** ***=*** ***10.2** **Hz, 2H), 1.68 (d, *J**** ***=*** ***12.9** **Hz, 2H), 1.12 (brs, 1H), 1.23–1.11 (m, 2H). ^13^** **C NMR (100** **MHz, DMSO-d_6_) δ 180.6, 167.4, 161.4, 157.9, 155.4, 153.8, 130.7, 122.6, 121.8, 115.6, 104.1, 99.9, 99.2, 65.9, 52.7, 52.6, 38.1, 28.6. HR-MS (ESI) Calcd for C_22_H_23_NO_6_ [M** **+** **H]^+^:398.1604, found: 398.1584.

#### 5,7-dihydroxy-3-(4-hydroxyphenyl)-6-(morpholinomethyl)-4H-chromen-4-one (1f).

White solid, yield: 16%; mp 210–212** **°C; ^1^H NMR (300** **MHz, DMSO-d_6_) *δ* 13.03 (s, 1H), 8.36 (s, 1H), 7.38 (d, *J**** ***=*** ***8.4** **Hz, 2H), 6.82 (d, *J**** ***=*** ***8.4** **Hz, 2H), 6.24 (s, 1H), 3.74 (s, 2H), 3.58 (m, 4H), 2.49 (m, 4H). ^13^C NMR (100** **MHz, DMSO-d_6_) *δ* 180.9, 164.6, 161.4, 157.9, 154.4, 143.3, 130.7, 122.7, 121.7, 115.6, 104.9, 100.7, 99.3, 66.6, 53.14, 51.4. HR-MS (ESI) Calcd for C_20_H_19_NO_6_ [M** **+** **H]^+^, 370.1291, found: 370.1320.

#### 5,7-dihydroxy-3-(4-hydroxyphenyl)-8-((4-methylpiperazin-1-yl)methyl)-4H-chromen-4-one (1g)

White solid, yield: 19%; mp 231–233** **°C; ^1^H NMR (300** **MHz, DMSO-d_6_) *δ* 8.34 (s, 1H), 7.37 (d, *J**** ***=*** ***8.4** **Hz, 2H), 6.82 (d, *J**** ***=*** ***8.7** **Hz, 2H), 6.18(s, 1H), 3.80(s, 2H), 3.55(m, 4H), 2.35 (m, 4H), 2.07(s, 3H). ^13^** **C NMR (100** **MHz, DMSO-d_6_) *δ* 180.7, 166.4, 161.5, 158.0, 155.6, 154.2, 130.7, 122.7, 121.7, 115.6, 104.7, 100.1, 99.6, 54.9, 52.4, 51.7, 46.0. HR-MS (ESI) Calcd for C_21_H_22_N_2_O_5_ [M** **+** **H]^+^, 383.1607, found: 383.1609.

#### 7-hydroxy-8-((4-(2-hydroxyethyl)piperazin-1-yl)methyl)-3-(4-hydroxyphenyl)-4H-chromen-4-one (2a)

White solid, yield: 20%; mp 230–232** **°C; ^1^H NMR (300** **MHz, DMSO-d_6_) δ 8.30 (s, 1H), 7.91 (d, *J**** ***=*** ***8.7** **Hz, 1H), 7.37(d, *J**** ***=*** ***8.7** **Hz, 2H), 6.88 (d, *J**** ***=*** ***9.0** **Hz, 1H), 6.77 (d, *J**** ***=*** ***8.4** **Hz, 2H), 3.95 (s, 2H), 3.49 (t, *J**** ***=*** ***6.3** **Hz, 2H), 2.57–2.48 (m, 8H), 2.38 (t, *J**** ***=*** ***6.3** **Hz, 2H). ^13^** **C NMR (100** **MHz, DMSO-d_6_) δ 175.3, 163.4, 157.7, 155.5, 153.0, 130.6, 126.3, 123.8, 123.0, 116.9, 115.5, 115.5, 108.7, 60.5, 59.0, 53.4, 52.7, 52.4. HR-MS (ESI) Calcd for C_22_H_24_N_2_O_5_ [M** **+** **H]^+^, 397.1763, found: 397.1767.

#### 7-hydroxy-8-((4-(hydroxymethyl)piperidin-1-yl)methyl)-3-(4-hydroxyphenyl)-4H-chromen-4-one (2b)

White solid, yield: 24%; mp 244–246** **°C; ^1^H NMR (300** **MHz, DMSO-d_6_) *δ* 8.28 (s, 1H), 7.89 (d, *J**** ***=*** ***8.7** **Hz, 1H), 7.37 (d, *J**** ***=*** ***8.4** **Hz, 2H), 6.84–6.77 (m, 3H), 3.98 (s, 2H), 3.26 (d, *J**** ***=*** ***6.3** **Hz, 2H), 2.95 (d, *J**** ***=*** ***11.1** **Hz, 2H), 2.21 (t, *J**** ***=*** ***11.1** **Hz, 2H), 1.73 (d, *J**** ***=*** ***13.2** **Hz, 2H), 1.43 (brs, 1H), 1.19 (m, 2H). ^13^** **C NMR (100** **MHz, DMSO-d_6_) *δ* 175.3, 164.2, 157.7, 155.5, 152.9, 130.6, 126.1, 123.8, 123.0, 116.6, 115.7, 115.5, 108.4, 66.00, 53.3, 52.9, 38.2, 28.9. HR-MS (ESI) Calcd for C_22_H_23_NO_5_ [M** **+** **H]^+^, 382.1654, found: 382.1648.

#### 7-hydroxy-3-(4-hydroxyphenyl)-8-((4-methylpiperidin-1-yl)methyl)-4H-chromen-4-one (2c)

White solid, yield: 14%; mp 250–252** **°C; ^1^H NMR (300** **MHz, DMSO-d_6_) *δ* 8.28 (s, 1H), 7.89 (d, *J**** ***=*** ***8.7** **Hz, 1H), 7.37 (d, *J**** ***=*** ***8.7** **Hz, 2H), 6.84–6.77 (m, 3H), 3.97 (s, 2H), 2.95 (d, *J**** ***=*** ***11.1** **Hz, 2H), 2.22 (t, *J**** ***=*** ***11.4** **Hz, 2H), 1.68 (d, *J**** ***=*** ***13.2** **Hz, 2H), 1.42 (brs, 1H), 1.17 (m, 2H), 0.91 (d, *J**** ***=*** ***6.6** **Hz, 3H). ^13^** **C NMR (100** **MHz, DMSO-d_6_) *δ* 175.3, 164.2, 157.7, 155.5, 152.9, 130.6, 126.1, 123.8, 123.0, 116.6, 115.7, 115.5, 108.5, 53.1, 34.1, 30.2, 22.0. HR-MS (ESI): Calcd for C_22_H_23_NO_4_ [M** **+** **H]^+^, 366.1705, found: 366.1749.

#### 8-(((3R,5S)-3,5-dimethylmorpholino)methyl)-7-hydroxy-3–(4-hydroxyphenyl)-4H-chromen-4-one (2d)

White solid, yield: 27%; mp 230–232** **°C; ^1^H NMR (300** **MHz, DMSO-d_6_) *δ* 8.32 (s, 1H), 7.92 (d, *J**** ***=*** ***9** **Hz, 1H), 7.38 (d, *J**** ***=*** ***9** **Hz, 2H), 6.93 (d, *J**** ***=*** ***9** **Hz, 1H), 6.80 (d, *J**** ***=*** ***8.4** **Hz, 2H), 3.88 (s, 2H), 3.56 (t, *J**** ***=*** ***8.4** **Hz, 2H), 2.83 (d, *J**** ***=*** ***10.8** **Hz, 2H), 1.90 (t, *J**** ***=*** ***11.1** **Hz, 2H), 1.05 (d, *J**** ***=*** ***6.3** **Hz, 6H). ^13^** **C NMR (100** **MHz, DMSO-d_6_) *δ* 175.4, 162.8, 157.8, 155.8, 153.1, 130.6, 126.4, 123.8, 123.00, 117.1, 115.5, 115.4, 109.1, 71.5, 58.7, 51.8, 19.4. HR-MS (ESI) Calcd for C_22_H_23_NO_5_ [M** **+** **H]^+^, 382.1654, found: 382.1664.

#### 7-hydroxy-3-(4-hydroxyphenyl)-8-((3-hydroxypiperidin-1-yl)methyl)-4H-chromen-4-one (2e)

White solid, yield: 15%; mp 226–228** **°C; ^1^H NMR (300** **MHz, DMSO-d_6_) *δ* 8.28 (s, 1H), 7.89 (d, *J**** ***=*** ***9** **Hz, 1H), 7.37 (d, *J**** ***=*** ***8.4** **Hz, 2H), 6.84–6.77 (m, 3H), 3.96 (s, 2H), 2.87 (d, *J**** ***=*** ***7.2** **Hz, 2H), 2.13 (t, *J**** ***=*** ***11.1** **Hz, 1H), 1.87 (t, *J**** ***=*** ***10.8** **Hz, 1H), 1.69–1.42 (m, 4H), 0.90 (m, 1H), 0.84 (d, *J**** ***=*** ***6.3** **Hz, 3H). ^13^** **C NMR (100** **MHz, DMSO-d_6_) δ 175.3, 164.2, 157.6, 155.4, 152.9, 130.6, 126.2, 123.8, 123.0, 116.6, 115.7, 115.5, 108.3, 60.6, 53.4, 53.2, 32.3, 31.2, 25.2, 19.7. HR-MS (ESI) Calcd for C_22_H_23_NO_4_ [M** **+** **H]^+^, 366.1705, found: 366.1716.

#### 7-hydroxy-3-(4-hydroxyphenyl)-8-(pyrrolidin-1-ylmethyl)-4H-chromen-4-one (2f).

White solid, yield: 23%; mp 177–179** **°C; ^1^H NMR (300** **MHz, DMSO-d_6_) *δ* 8.27 (s, 1H), 7.88 (d, *J**** ***=*** ***8.7** **Hz, 1H), 7.37 (d, *J**** ***=*** ***8.4** **Hz, 2H), 6.84–6.77 (m, 3H), 4.08 (s, 2H), 2.67 (m, 4H), 1.77 (m, 4H). ^13^C NMR (100** **MHz, DMSO-d_6_) *δ* 175.3, 164.7, 157.7, 155.4, 152.8, 130.6, 126.2, 123.8, 123.1, 116.1, 115.9, 115.5, 109.1, 53.6, 50.0, 23.7. HR-MS (ESI) Calcd for C_20_H_19_NO_4_ [M** **+** **H]^+^, 338.1392, found: 338.1413.

#### (S)-7-hydroxy-8-((2-(hydroxymethyl)pyrrolidin-1-yl)methyl)-3–(4-hydroxyphenyl)-4H-chromen-4-one (2g)

White solid, yield: 35%; mp 205–207** **°C; ^1^H NMR (300** **MHz, DMSO-d_6_) *δ* 8.27 (s, 1H), 7.88 (d, *J**** ***=*** ***8.7** **Hz, 1H), 7.37 (d, *J**** ***=*** ***8.7** **Hz, 2H), 6.85–6.77 (m, 3H), 4.34–4.01 (s, 2H), 3.51 (brs, 2H), 2.92–2.83 (d, *J**** ***=*** ***27.6** **Hz, 2H), 2.40 (d, *J**** ***=*** ***8.1** **Hz, 1H), 1.89 (m, 1H), 1.67 (m, 3H). ^13^** **C NMR (100** **MHz, DMSO-d_6_) *δ* 175.3, 172.8, 157.8, 155.2, 152.6, 138.2, 130.6, 126.1, 123.8, 123.0, 120.0, 115.5, 109.6, 65.6, 62.8, 54.6, 49.5, 27.6, 23.1. HR-MS (ESI) Calcd for C_21_H_21_NO_5_ [M** **+** **H]^+^, 368.1498, found: 368.1482.

#### 7-hydroxy-3-(4-hydroxyphenyl)-8-((2-methylpiperidin-1-yl)methyl)-4H-chromen-4-one (2h)

White solid, yield: 12%; mp 228–230** **°C; ^1^H NMR (300** **MHz, DMSO-d_6_) *δ* 8.28 (s, 1H),7.89 (d, *J**** ***=*** ***8.7** **Hz, 1H), 7.37 (d, *J**** ***=*** ***8.4** **Hz, 2H), 6.79–6.77 (m, 3H), 4.32–4.27 (d, *J**** ***=*** ***15, 1H), 3.9–3.85 (d, *J**** ***=*** ***15** **Hz, 1H), 2.83 (d, *J**** ***=*** ***12** **Hz, 1H), 2.66 (brs, 1H), 2.30 (t, *J**** ***=*** ***9.3** **Hz, 1H), 1.62–1.35 (m, 6H), 1.15 (d, *J**** ***=*** ***6.3** **Hz, 3H). ^13^** **C NMR (100** **MHz, DMSO-d_6_) δ 175.3, 164.6, 157.7, 155.2, 152.9, 130.6, 125.9, 123.8, 123.1, 116.4, 115.9, 115.4, 108.7, 57.8, 56.3, 50.4, 36.3, 33.8, 25.6, 22.5. HR-MS (ESI) Calcd for C_22_H_23_NO_4_ [M** **+** **H]^+^, 366.1705, found: 366.1731.

#### 7-hydroxy-3-(4-hydroxyphenyl)-8-((4-methylpiperazin-1-yl)methyl)-4H-chromen-4-one (2i)

White solid, yield: 18%; mp 215–217** **°C; ^1^H NMR (300** **MHz, DMSO-d_6_) *δ* 8.28 (s, 1H), 7.90 (d, *J**** ***=*** ***8.7** **Hz, 1H), 7.37 (d, *J**** ***=*** ***8.7** **Hz, 2H), 6.88–6.77 (m, 3H), 3.93(s, 2H), 2.56 (m, 4H), 2.34 (m, 4H), 2.15 (s, 3H). ^13^** **C NMR (100** **MHz, DMSO-d_6_) *δ* 175.4, 163.3, 157.7, 155.6, 153.1, 137.2, 130.6, 126.3, 123.9, 123.0, 116.9, 115.5, 108.9, 55.0, 52.6, 52.3, 46.1. HR-MS (ESI) Calcd for C_21_H_22_N_2_O_4_ [M** **+** **H]^+^, 367.1658, found: 367.1646.

#### 7-hydroxy-3-(4-methoxyphenyl)-8-((4-methylpiperazin-1-yl)methyl)-4H-chromen-4-one (2j)

White solid, yield: 23%; mp 202–204** **°C; ^1^H NMR (300** **MHz, DMSO-d_6_) *δ* 8.36 (s,1H), 7.92 (d, *J**** ***=*** ***8.7** **Hz, 1H), 7.50 (d, *J**** ***=*** ***9** **Hz, 2H), 6.99 (d, *J**** ***=*** ***8.7** **Hz, 2H), 6.87 (d, *J**** ***=*** ***9** **Hz, 1H), 3.95 (s, 2H), 3.77 (s, 3H), 2.57 (m, 4H), 2.36 (m, 4H), 2.16 (s, 3H). ^13^C NMR (100** **MHz, DMSO-d_6_) *δ* 175.3, 163.2, 159.5, 155.6, 153.4, 130.6, 126.3, 124.7, 123.5, 116.9, 115.5, 114.1, 109.0, 55.7, 55.0, 52.6, 52.3, 46.1. HR-MS (ESI) Calcd for C_22_H_24_N_2_O_4_ [M** **+** **H]^+^, 381.1814, found: 381.1814.

#### 7-hydroxy-3-(4-methoxyphenyl)-8-(morpholinomethyl)-4H-chromen-4-one (2k).

White solid, yield: 28%; mp 235–237** **°C; ^1^H NMR (300** **MHz, DMSO-d_6_) *δ* 8.31 (s, 1H), 7.92 (d, *J**** ***=*** ***8.7** **Hz, 1H), 7.38 (d, *J**** ***=*** ***8.7** **Hz, 2H), 6.93 (d, *J**** ***=*** ***9** **Hz, 1H), 6.77 (d, *J**** ***=*** ***8.4** **Hz, 2H), 3.88 (s, 3H), 3.59 (m, 6H), 2.48 (m, 4H). ^13^C NMR (100** **MHz, DMSO-d_6_) *δ* 175.4, 162.6, 157.7, 155.9, 153.2, 130.6, 126.4, 123.8, 123.0, 117.1, 115.5, 115.3, 109.3, 66.6, 53.2, 52.0. HR-MS (ESI) Calcd for C_20_H_19_NO_5_ [M** **+** **H]^+^, 354.1341, found: 354.1315.

#### 7-hydroxy-3-(4-methoxyphenyl)-8-((4-methylpiperidin-1-yl)methyl)-4H-chromen-4-one (2l)

White solid, yield: 21%; mp 208–210** **°C; ^1^H NMR (300** **MHz, DMSO-d_6_) *δ* 8.33 (s, 1H), 7.90 (d*, J**** ***=*** ***8.7** **Hz, 1H), 7.50 (d, *J**** ***=*** ***9** **Hz, 2H), 6.95 (d, *J**** ***=*** ***11.7** **Hz, 2H), 6.82 (d, *J**** ***=*** ***9** **Hz, 1H), 3.98 (s, 2H), 3.77 (s, 3H), 2.96 (d, *J**** ***=*** ***11.4** **Hz, 2H), 2.22 (t, *J**** ***=*** ***10.8** **Hz, 2H), 1.68 (d, *J**** ***=*** ***12.3** **Hz, 2H), 1.42 (brs, 1H), 1.17 (m, 2H), 0.91 (d, *J**** ***=*** ***6.6** **Hz, 3H). ^13^C NMR (100** **MHz, DMSO-d_6_) *δ* 175.2, 164.3, 159.5, 155.5, 153.2, 130.6, 126.2, 124.7, 123.5, 116.6, 115.8, 114.1, 108.4, 55.7, 53.2, 53.1, 34.1, 30.2, 22.0. HR-MS (ESI) Calcd for C_23_H_25_NO_4_ [M** **+** **H]^+^, 380.1862, found: 380.1881.

#### 7-hydroxy-3-(4-methoxyphenyl)-8-(pyrrolidin-1-ylmethyl)-4H-chromen-4-one (2m)

White solid, yield: 12%; mp 173–175** **°C; ^1^H NMR (300** **MHz, DMSO-d_6_) *δ* 8.32 (s, 1H),7.89 (d, *J**** ***=*** ***8.7** **Hz, 1H), 7.50 (d, *J**** ***=*** ***8.7** **Hz, 2H), 6.98 (d, *J**** ***=*** ***8.7** **Hz, 2H),6.82 (d, *J**** ***=*** ***8.7** **Hz, 1H), 4.09 (s, 2H), 3.77 (s, 3H), 2.68 (m, 4H), 1.77 (m, 4H). ^13^** **C NMR (100** **MHz, DMSO-d_6_) *δ* 175.2, 164.5, 159.5, 155.4, 153.1, 130.6, 126.2, 124.8, 123.4, 116.2, 115.9, 114.1, 109.3, 55.6, 53.6, 49.9, 23.7. HR-MS (ESI) Calcd for C_21_H_21_NO_4_ [M** **+** **H]^+^, 352.1549, found: 352.1568.

#### 7-hydroxy-8-((4-hydroxypiperidin-1-yl)methyl)-3–(4-methoxyphenyl)-4H-chromen-4-one (2n)

White solid, yield: 21%; mp 205–207 °C; ^1^H NMR (300** **MHz, DMSO-d_6_) *δ* 8.33 (s, 1H), 7.90 (d, *J**** ***=*** ***8.7** **Hz, 1H), 7.50 (d, *J**** ***=*** ***8.7** **Hz, 2H), 6.98 (d, *J**** ***=*** ***9** **Hz, 2H), 6.83 (d, *J**** ***=*** ***9** **Hz, 1H), 3.96 (s, 2H), 3.77 (s, 3H), 3.56 (brs, 1H), 2.80 (t, *J**** ***=*** ***7.2** **Hz, 2H), 2.35 (t, *J**** ***=*** ***10.8** **Hz, 2H), 1.75 (d, *J**** ***=*** ***12.9** **Hz, 2H), 1.45 (q, *J**** ***=*** ***6.9** **Hz, 2H). ^13^** **C NMR (100** **MHz, DMSO-d_6_) *δ* 175.2, 164.1, 159.5, 155.4, 153.2, 131.5, 125.9, 124.3, 123.4, 116.5, 115.3, 113.9, 109.4, 55.6, 55.4, 52.9, 50.6, 34.4. HR-MS (ESI) Calcd for C_22_H_23_NO_5_ [M** **+** **H]^+^, 382.1654, found: 382.1669.

#### 7-hydroxy-3-(4-methoxyphenyl)-8-((3-methylpiperidin-1-yl)methyl)-4H-chromen-4-one (2o)

White solid, yield: 21%; mp 165–167** **°C; ^1^H NMR (300** **MHz, DMSO-d_6_) *δ* 8.33 (s, 1H), 7.90 (d, *J**** ***=*** ***9** **Hz, 1H), 7.50 (d, *J**** ***=*** ***8.7** **Hz, 2H), 6.98 (d, *J**** ***=*** ***9** **Hz, 2H), 6.82 (d, *J**** ***=*** ***9** **Hz, 1H), 3.96 (s, 2H), 3.77 (s, 3H), 2.87 (d, *J**** ***=*** ***7.5** **Hz, 2H), 2.17(t, *J**** ***=*** ***11.7** **Hz, 1H), 1.83 (t, *J**** ***=*** ***10.8** **Hz, 1H), 1.65–1.49 (m, 4H), 0.93 (m, 1H), 0.82 (d, *J**** ***=*** ***6.6** **Hz, 3H). ^13^C NMR (100** **MHz, DMSO-d_6_) *δ* 175.2, 164.3, 159.5, 155.5, 153.2, 130.6, 126.2, 124.7, 123.5, 116.6, 115.7, 114.1, 108.4, 60.6, 55.7, 53.4, 53.2, 32.3, 31.2, 25.2, 19.7. HR-MS (ESI) Calcd for C_23_H_25_NO_4_ [M** **+** **H]^+^, 380.1862, found: 380.1897.

#### 7-hydroxy-8-((3-hydroxypiperidin-1-yl)methyl)-3–(4-methoxyphenyl)-4H-chromen-4-one (2p)

White solid, yield: 25%; mp 188–190** **°C; ^1^H NMR (300** **MHz, DMSO-d_6_) *δ* 8.33 (s, 1H), 7.91 (d, *J**** ***=*** ***9** **Hz, 1H), 7.50 (d, *J**** ***=*** ***8.7** **Hz, 2H), 6.98 (d, *J**** ***=*** ***9** **Hz, 2H), 6.84 (d, *J**** ***=*** ***8.7** **Hz, 1H), 3.95 (s, 2H), 3.77 (s, 3H), 3.58 (brs, 1H), 2.85 (d, *J**** ***=*** ***9.3** **Hz, 1H), 2.66 (d, *J**** ***=*** ***11.1** **Hz, 1H), 2.27–2.13 (m, 2H), 1.71 (d, *J**** ***=*** ***10.5** **Hz, 2H), 1.45 (m, 1H), 1.24 (m, 1H). ^13^C NMR (100** **MHz, DMSO-d_6_) *δ* 175.2, 164.0, 159.5, 155.5, 153.2, 130.6, 126.2, 124.7, 123.5, 116.7, 115.7, 114.1, 108.6, 65.8, 60.4, 55.6, 53.0, 52.9, 32.6, 22.7. HR-MS (ESI) Calcd for C_22_H_23_NO_5_ [M** **+** **H]^+^, 382.1654, found:382.1718.

#### 8-((4-benzylpiperazin-1-yl)methyl)-7-hydroxy-3-(4-methoxyphenyl)-4H-chromen-4-one (2q)

White solid, yield: 27%; mp 220–222** **°C; ^1^H NMR (300** **MHz, DMSO-d_6_) *δ* 8.36 (s,1H), 7.92 (d, *J**** ***=*** ***8.7** **Hz, 1H), 7.50 (d, *J**** ***=*** ***8.7** **Hz, 2H), 7.29 (m, 5H), 6.98 (d, *J**** ***=*** ***8.7** **Hz, 2H), 6.87 (d, *J**** ***=*** ***8.7** **Hz, 1H), 3.96 (s, 2H), 3.77 (s, 3H), 3.47 (s, 2H), 2.59–2.49 (m, 8H). ^13^C NMR (100** **MHz, DMSO-d_6_) *δ* 175.3, 159.7, 159.5, 157.4, 155.7, 142.5, 138.5, 130.6, 129.4, 128.7, 127.3, 124.4, 123.3, 116.7, 115.5, 114.1, 108.6, 69.1, 62.4, 55.8, 52.9, 52.6. HR-MS (ESI) Calcd for C_28_H_28_N_2_O_4_ [M** **+** **H]^+^, 451.2127, found: 457.2113.

#### 7-hydroxy-8-((4-(hydroxymethyl)piperidin-1-yl)methyl)-3-(4-methoxyphenyl)-4H-chromen-4-one (2r)

White solid, yield: 27%; mp 195–197** **°C; ^1^H NMR (300** **MHz, DMSO-d_6_) *δ* 8.33 (s, 1H), 7.90 (d, *J**** ***=*** ***8.7** **Hz, 1H), 7.50 (d, *J**** ***=*** ***8.7** **Hz, 2H), 6.98 (d, *J**** ***=*** ***9** **Hz, 2H), 6.82 (d, *J**** ***=*** ***9** **Hz, 1H), 3.98 (s, 2H), 3.77 (s, 3H), 3.27 (d, *J**** ***=*** ***6** **Hz, 2H), 2.99 (d, *J**** ***=*** ***11.1** **Hz, 2H), 2.25 (t, *J**** ***=*** ***11.4** **Hz, 2H), 1.73 (d, *J**** ***=*** ***12.9** **Hz, 2H), 1.23 (brs, 1H), 1.11 (m, 2H). ^13^** **C NMR (100** **MHz, DMSO-d_6_) *δ* 175.2, 164.3, 159.5, 155.5, 153.2, 130.6, 126.2, 124.7, 123.5, 116.5, 115.8, 114.1, 108.4, 66.00, 55.7, 53.3, 52.9, 38.2, 28.9. HR-MS (ESI) Calcd for C_23_H_25_NO_5_ [M** **+** **H]^+^, 396.1811, found: 396.1806.

#### 7-hydroxy-8-((4-(2-hydroxyethyl)piperazin-1-yl)methyl)-3-(4-methoxyphenyl)-4H-chromen-4-one (2s)

White solid, yield: 27%; mp 196–198** **°C; ^1^H NMR (300** **MHz, DMSO-d_6_) *δ* 8.35 (s, 1H), 7.91 (d, *J**** ***=*** ***8.7** **Hz, 1H), 7.50 (d, *J**** ***=*** ***8.7** **Hz, 2H), 6.98 (d, *J**** ***=*** ***9** **Hz, 2H), 6.89 (d, *J**** ***=*** ***9** **Hz, 1H), 3.94 (s, 2H), 3.77 (s, 3H), 3.49 (t, *J**** ***=*** ***6.3** **Hz, 3H), 2.57–2.40 (m, 8H), 2.36 (t, *J**** ***=*** ***7.2** **Hz, 2H). ^13^C NMR (100** **MHz, DMSO-d_6_) *δ* 175.2, 163.4, 159.5, 155.6, 153.3, 130.6, 126.3, 124.7, 123.3, 116.9, 115.5, 114.1, 108.8, 60.5, 59.0, 55.7, 53.5, 52.7, 52.4. HR-MS (ESI) Calcd for C_23_H_26_N_2_O_5_ [M** **+** **H]^+^, 411.1920, found: 411.1904.

#### 7-hydroxy-3-(4-methoxyphenyl)-8-((2-methylpiperidin-1-yl)methyl)-4H-chromen-4-one (2t)

White solid, yield: 17%; mp 141–143 °C; ^1^H NMR (300** **MHz, DMSO-d_6_) *δ* 8.32 (s, 1H), 7.87 (d, *J**** ***=*** ***9.9** **Hz, 1H), 7.50 (d, *J**** ***=*** ***9.9** **Hz, 2H), 6.98 (d, *J**** ***=*** ***9** **Hz, 2H), 6.80 (d, *J**** ***=*** ***9.9** **Hz, 1H), 4.31–4.26 (d, *J**** ***=*** ***15** **Hz, 1H), 3.90–3.85 (d, *J**** ***=*** ***15** **Hz, 1H), 3.77 (s, 3H), 2.83 (d, *J**** ***=*** ***12.3** **Hz, 1H), 2.66 (brs, 1H), 2.33 (t, *J**** ***=*** ***9.6** **Hz, 1H), 1.48–1.35 (m, 6H), 1.15 (d, *J**** ***=*** ***6.3** **Hz, 3H). ^13^C NMR (100** **MHz, DMSO-d_6_) *δ* 175.1, 164.8, 159.5, 155.2, 153.1, 130.6, 125.9, 124.8, 123.5, 116.4, 115.9, 114.1, 108.6, 56.6, 55.6, 51.7, 50.4, 33.8, 26.9, 25.7, 22.6. HR-MS (ESI) Calcd for C_23_H_25_NO_4_ [M** **+** **H]^+^, 380.1862, found: 380.2130.

## Bioassay studies

### Cell lines and cell culture

The cell-based histamine receptor 3 (H_3_R) assay was carried out based on β-lactamase complementation technology. The H3-bla U2OS cells (Invitrogen, Invitrogen, Waltham, Massachusetts) stably expressed two fusion proteins, as well as a β-lactamase reporter gene under the control of a UAS response element. The first fusion protein was human H_3_R linked to a Gal4-VP16 transcription factor through the TEV protease site, and the other was the β-arrestin/TEV protease fusion protein. H_3_-bla U2OS cells were cultured in McCoy^’^s 5** **A Medium supplemented with 10% foetal bovine serum (FBS; Gibco, Shanghai, China) at 37** **°C in a humidified atmosphere with 5% CO_2_. To each well in a 384-well plate was seeded exponentially growing cells in a density of 6.5** **×** **10^3^ cells/mL in 32** **μL of media. The plate was incubated at 37** **°C, 18–24** **h, 5% CO_2_ for cell adherence.

### Fluorescent H_3_R assay

Stock solutions of test compounds (10** **mM) were prepared in DMSO and then diluted 100 times in media. Cells were exposed to 4** **μL of test compounds and the control compound thioperamide (Sigma-Aldrich, St. Louis, Missouri) for 30** **min and then stimulated with 4** **μL of methylhistamine at 400** **nM (Sigma-Aldrich) for 5** **h. Then, 8** **μL of LiveBLAzer-FRET B/G Substrate (CCF4-AM; Invitrogen) was added and incubation continued for 2** **h. Plates were subjected to the fluorescence reading with a Spectra Max M5 microplate reader (BioTek, Winooski, Vermont); equipped with 410** **nm excitation and 460** **nm and 530** **nm emission filters. The inhibition percentage was calculated based on the fluorescence according to the following equation: % inhibition = (Model_Response ratio_–Compound_Response ratio_)/Model_Response ratio_. And IC_50_ values were determined from log concentration − inhibition curves. At least three separate tests were carried out.

### Molecular docking

We chose the most active compounds for molecular docking studies to predict how molecules and proteins work. A homology modelling of H_3_R was built as our previous report^25^. The 3D structure of compound **2h** was built using DS MODELER (Discovery Studio 2016, BIOVIA Inc, San Diego, CA) and evaluated the model according to the PDF Total Energy and the Profile-3D procedure. Flexible Docking was used for the docking procedure. The 3D model of H_3_R with the lowest PDF Total Energy was chosen for docking. Water and the cognate ligand (doxepin) were removed from the model, and hydrogen atoms were added to amino acid residues. The binding mode was shown by DS visualizer.

## Results and discussion

### Structure–activity relationship

**Figure 2.**
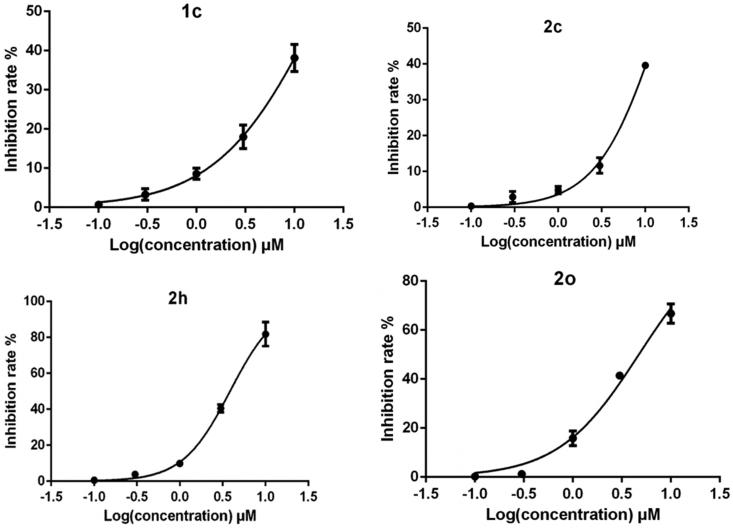
The IC_50_ of the four compounds (**1c**, **2c**, **2h**, and **2o**) showed good H_3_R inhibitory activity.

The compounds were initially evaluated for inhibition rate on H_3_R at a fixed concentration of 10** **μM (Tables 1 and 2). Of the 27 compounds evaluated, four compounds （**1c**, **2c**, **2h**, **2o**）performed satisfactory inhibitory effect (Figure 2). According to reports in the literature, H_3_R inhibitory activities were increased by the introduction of pyrrolidine and piperidine to the iso-flavone scaffold^10^. Thus, we introduced various pyrrolidine, piperidine, piperazine and morpholine moieties onto 6- or 8-position of iso-flavone. The results for series 1 are shown in Table 1. The advantage of piperidine groups outweighed pyrrolidine moieties. As for substituted piperazine and morpholine moieties, the subsequent data did not give satisfactory results. Then, we modified daidzein and formononetin with substituted piperidine and pyrrolidine fragments. It should be noted that further steric modification on piperidine was detrimental for the inhibitory activities. For example, 4-hydroxymethyl, 3-hydroxy piperidine (compound **2b**, **2e**) attached to the structure of daidzein led the inhibitory activity to decrease. However, the 2-methyl piperidine group (compound **2h**) showed very strong inhibition. Interestingly, for formononetin, 3-methyl piperidine (compound **2o**) and pyrrolidine (compound **2m**) fragments showed unexpected inhibitory effect. Structurally, substituted piperidine (such as methyl- and hydroxyl-) or pyrrolidine groups could improve bioactivity but bulky substitutions may hinder binding H_3_ pockets, namely, binding affinity would loss^10^. Comparing different iso-flavone structures, even though 4′-hydroxy or 4′-methoxy benzene ring in 4-position of iso-flavone scaffold showed significant fluctuation in bioactivity level according to the data shown in Table 2, in most cases, daidzein derivatives have advantages over formononetin as H_3_R antagonists, for example, compound **2c** vs **2l**; **2h** vs **2t**.

**Table 1.** Structures and activities of compounds **1a**–**1g**.


Compound	R_1_	R_2_	Inhibit rate (%) at 10 μM	IC_50_ (μM)
**1a**		**H**	–7.61	
**1b**		**H**	–10.31	
**1c**		**H**	**38.14**	**17.83 ± 0.06**
**1d**	**H**		–61.00	
**1e**		**H**	–7.54	
**1f**	**H**		–19.15	
**1g**		**H**	–43.72	
**Thioperamide**			**72.34**	1.03 ± 0.01

Bold values indicates that the compound has a high inhibit rate (%) at 10 µM and is able to posses an IC50.

**Table 2.** Structures and activities of compounds **2a**–**2t**.


Compound	R_3_	R_4_	Inhibit rate (%) at 10 μM	IC_50_ (μM)	Compd.	R_3_	R_4_	Inhibit rate (%) at 10 μM	IC_50_ (μM)
**2a**		**OH**	–1.85		**2k**		**OCH_3_**	–23.17	
**2b**		**OH**	–32.59		**2l**		**OCH_3_**	–1.51	
**2c**		**OH**	**39.59**	**14.24 ± 0.08**	**2m**		**OCH_3_**	14.56	
**2d**		**OH**	–57.52		**2n**		**OCH_3_**	1.72	
**2e**		**OH**	–0.82		**2o**		**OCH_3_**	**66.77**	**4.71 ± 0.01**
**2f**		**OH**	18.72		**2p**		**OCH_3_**	–9.49	
**2g**		**OH**	–8.12		**2q**		**OCH_3_**	–9.56	
**2h**		**OH**	**81.83**	**3.84 ± 0.04**	**2r**		**OCH_3_**	–55.09	
**2i**		**OH**	–20.42		**2s**		**OCH_3_**	–42.75	
**2j**		**OCH_3_**	–9.69		**2t**		**OCH_3_**	2.53	

Bold values indicates that the compound has a high inhibit rate (%) at 10 μM and is able to posses an IC50.

### Binding modes of compound 2h

**Figure 3.**
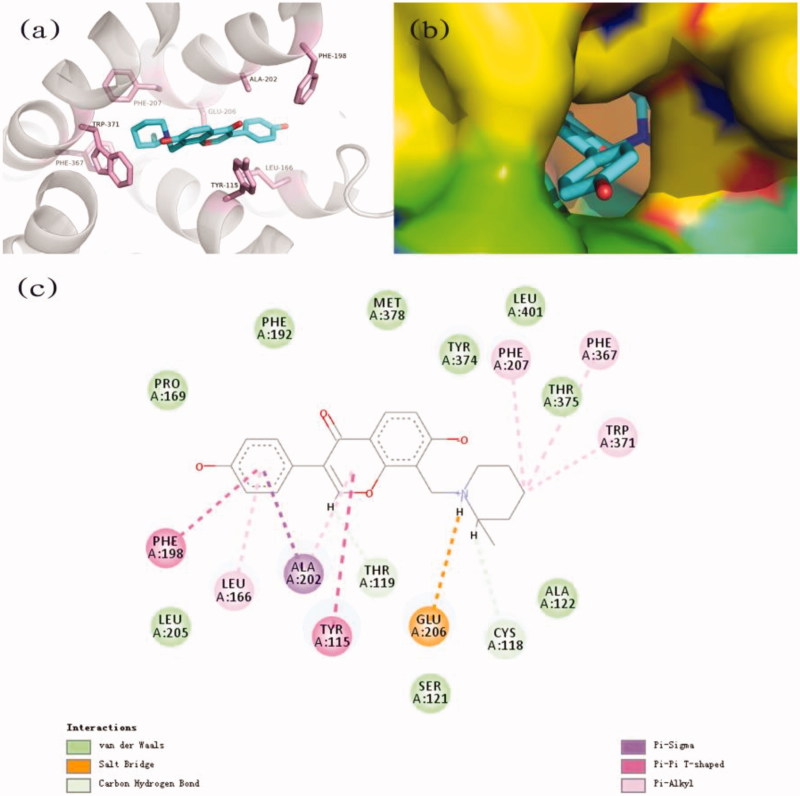
(a) The predicted binding mode of compound **2h** with H_3_R; (b) the binding pocket of H_3_R by the surface representation; (c) 2D schematic diagram of potential interactions between compound **2h** and H_3_R.

The results showed that compound **2h** bound with H_3_R through multiple sites (Figure 3). The protonated amine of the pyridine group interacted with Glu206 through a salt bridge. The Tyr-115 and Phe-198 bound to the aromatic ring structural on one side of compound **2h** by π–π T-shape interactions. In addition to this, compound **2h** also formed hydrophobic interaction, π–sigma and π–alkyl interaction with the protein.

## Conclusions

In this work, two series of iso-flavone derivatives were synthesised and evaluated for their H_3_R inhibitory activity. Ultimately, we identified compound **1c**, **2c**, **2h**, **2o** which possessed favourable H_3_R inhibitory activity. The structure–activity relationship (SAR) study identified the piperazine group in the 8-position of iso-flavone was essential for the H_3_R inhibitory activity (compound **2h**). Molecular docking showed 2′-methyl piperidine substituent of **2h** formed a salt bridge and hydrophobic interactions with the protein. In this paper, we creatively modified the iso-flavone derivatives and determined this scaffold possessing the potential H_3_R inhibitory activity. Moreover, these results also provided clues for the development of novel H_3_R antagonists.
